# Bridging multiscale dynamics with AIE: a case study in polymer emulsions

**DOI:** 10.1093/nsr/nwaf494

**Published:** 2025-11-10

**Authors:** Bo Chu, Bin Liu

**Affiliations:** Department of Chemical and Biomolecular Engineering, National University of Singapore, Singapore; Department of Chemical and Biomolecular Engineering, National University of Singapore, Singapore

**Figure 1.**
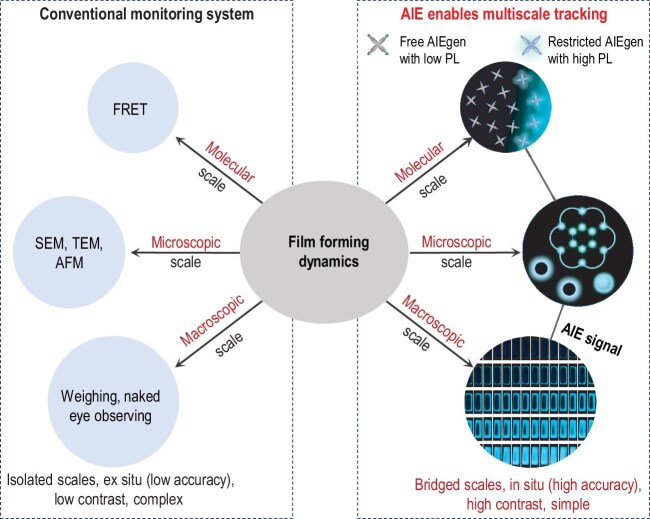
Multiscale tracking strategy integrating molecular, microscopic, and macroscopic approaches, comparing conventional (left) and AIE-enabled (right) monitoring systems.

Complex multiscale dynamics are fundamental to materials, with the interplay across molecular, microscopic, and macroscopic levels being crucial for understanding their underlying mechanisms. Conventional monitoring approaches, however, often rely on multiple technologies, posing challenges in data integration and limiting spatiotemporal resolution [1]. Using polymer emulsions as a model system (Fig. 1), Profs Ben Zhong Tang, Xianhong Wang, and coauthors addressed these limitations by employing high-contrast fluorescence activated through restricted intramolecular motion [2]. This pioneering aggregation-induced emission (AIE)-based strategy enabled the simultaneous correlation of multiscale dynamics within a unified optical framework, providing deep and comprehensive insights into the behavior of complex systems.

Polymer emulsions, widely utilized in coatings and adhesives, exhibit intricate multiscale dynamics during film formation, encompassing water evaporation, particle coalescence, and polymer diffusion [3,4]. Traditional techniques such as fluorescence resonance energy transfer (FRET) or electron microscopy are limited by their scale-specificity, low contrast, complex sample preparation, and inefficiency [5,6]. A unified, real-time method capable of bridging these scales is essential to overcome these limitations.

To address these challenges, Tang and coauthors [2] employed water-soluble AIEgens as fluorescent probes. At the molecular level, the fluorescence emission of AIEgens was activated by restricted intramolecular motion during water evaporation, enabling real-time monitoring of molecular transitions from free movement to restricted states. At the microscopic scale, structured illumination microscopy captured dynamic processes such as particle coalescence and capillary-driven fusion, revealing critical drying behaviors. At the macroscopic scale, high-contrast fluorescence imaging tracked the progression of drying fronts and film thickening, offering detailed insights into the film-forming process. This multiscale strategy successfully bridged molecular, microscopic, and macroscopic regimes within a single fluorescence-based platform.

The industrial relevance of this methodology was demonstrated in the coatings industry, where AIEgens were integrated into waterborne wood coatings to monitor drying dynamics over large surfaces. Real-time fluorescence imaging effectively visualized the drying process, distinguishing between wet, particle-packing, and fully dried regions. Grayscale analysis of fluorescence images enabled precise, cost-efficient monitoring without the need for complex instrumentation, underscoring the method’s practicality for industrial applications.

Using polymer emulsions as a case study, Tang and coauthors [2] established a robust framework for multiscale analysis, highlighting the transformative potential of AIE technology in bridging cross-scale material dynamics. Beyond polymer emulsions, this methodology offers broad applicability in diverse fields, including life sciences (e.g. neural transmission and wound healing) and materials science (e.g. battery dynamics and corrosion monitoring). By unifying multiscale insights within a single optical framework, this work sets the stage for more efficient, accurate, and generalizable process analysis in complex systems.
